# Model-Predictive Control for the Three-Tank System Utilizing an Industrial Automation System

**DOI:** 10.1021/acsomega.2c01275

**Published:** 2022-05-20

**Authors:** Jukka Kortela

**Affiliations:** Department of Chemical and Metallurgical Engineering, Aalto University School of Chemical Engineering, P.O. Box 16100, FI-00076 Aalto, Finland

## Abstract

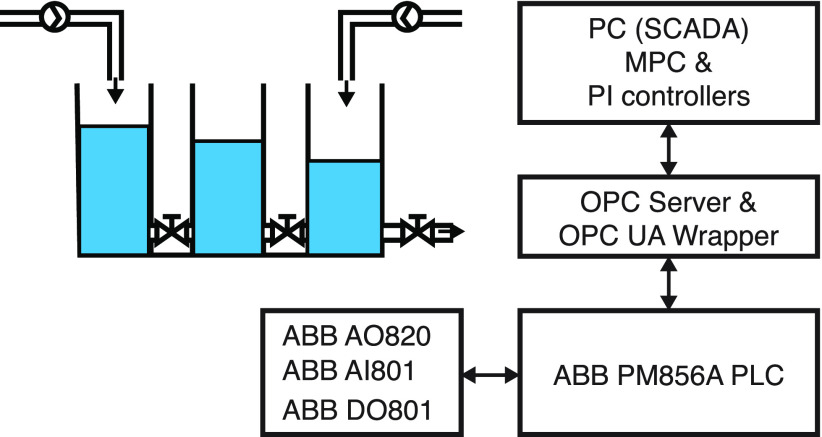

A three-tank process has difficulty in controller design because of nonlinear flow and interactions between tanks. This paper addresses the design methodology of the model-predictive controller (MPC) for the three-tank system. The control performance of the proposed MPC controller is compared with the proportional plus integral (PI) controller by both simulations and experiments on the real three-tank pilot with the industrial ABB 800xA automation system. The MPC controller shows a faster response for the two tanks: In the simulation, the settling times are about 120 s for both tanks of the MPC controller. On the other hand, the settling times for the PI controller are about 200 s for the first tank and 150 s for the second tank. The experiments confirm these results.

## 1 Introduction

Typical processes require the simultaneous control of several variables related to one system. Each input may affect all system outputs. The liquid level is one of the important controlled variables in modern process control, and control accuracy plays an important role in improving product quality and enhancing economic benefits. The three-tank system is a typical multivariable system with features of strong coupling and nonlinearity, which gives it great research value in the study of liquid level control.^1^

Many control methods have been proposed for the liquid level tracking control problem of the three-tank system. An interval type-II fuzzy logic systems (IT2FLS) is presented by Sahu and Ayyagari,^2^ and it is compared with a linear quadratic regulator (LQR). The test results show that the response is oscillatory when the liquid level is controlled by the LQR controller. In contrast, IT2FLC can achieve a much faster and better response. A method for a rough controller based on rough set theory (RST) was proposed by Aixian and Yun^3^ for the control of the liquid levels of the three-tank system. The key element in designing a rough controller is extracting rule sets from human behavior data according to the RST algorithm. The results show that the method of the rough controller (RC) is feasible, and the control performance is satisfactory.

The simplest form of the coupled multivariable system of the level control is the two-tank system with 2 inputs and 2 outputs presented by Essahafi.^4^ First a state space model is developed for the system. Then the unconstrained model-predictive control (MPC) is designed. The simulation results show that the MPC controller allows a good disturbance rejection and robustness. di Capaci et al.^5^ present three different formulations of MPC to handle static friction in control valves. The quadruple-tank process is used as a testing simulation environment. It is observed that stiction embedding nonlinear MPC only can guarantee good performance in set-points tracking and also stiction compensation. Piñón et al.^6^ validate the multiple-input multiple-output adaptive predictive controller (MIMO-APC) with the two simulated processes: a quadrotor drone and the quadruple-tank process. The simulation shows excellent set-point tracking behavior in the quadruple tank, in comparison to that with the control strategies previously reported in the literature.

Nonlinear model predictive control (NMPC) uses a more accurate nonlinear model for the control of the three-tank system.^7^ An experimental stability study of NMPC was carried out on the quadruple-tank process by Raff et al.^8^ The results showed that NMPC does not naturally guarantee closed loop stability. The closed loop asymptotic stability can be achieved with the NMPC approaches developed in theory. Yu et al.^9^ developed a controller composed of a feed-forward and feed-back controller for the three-tank systems. An improved cuckoo algorithm is proposed to solve the optimization problem involved in the developed nonlinear model predictive control. However, measurement and model mismatches lead to large errors in the experiment. In the nonconvex problem, an optimal solution cannot be guaranteed. In addition, an optimization problem can be too large to be solved online. A novel algorithm for utilizing the bees algorithm in an MPC is proposed by Sarailoo et al.^10^ in order to control a class of nonlinear systems. However, the computational burden is still too heavy to implement.

MPC is able to handle constraints in the MIMO systems and attenuate disturbances since the optimization problem is solved online with new measurements. Compared with, for example, the PID controller, MPC can achieve a faster response and no overshoot.^11^ In order to show the effectiveness of the MPC controller, the control performance of the proposed MPC controller is compared with the PI controller by both simulation and experiments. For the PI controller, there are well-established methods for tuning and stability analysis. Therefore, it is used in comparison. The main contributions of this paper are summarized as follows: (1) The nonlinear model of the three-tank system is presented, and the parameters of the simulation model are identified as close as possible to model the real system used in the experimental setup. (2) The state-space-based MPC is implemented by taking into account the computational burden. (3) The parameters of PI controllers are tuned and detuned with the well-established methods. In addition, the performance of the MPC and PI controllers are evaluated with the time-integral performance criteria. (4) The test results are validated on an experimental benchmark using the industrial automation technology with a new OPC UA communication technology where the advanced control is implemented on a remote computer independent of the used automation system.

This paper presents a MPC for the three-tank system. The paper is organized as follows: section 2 presents the dynamic models of the three-tank system. Section 3 presents the MPC controller for the three-tank system. The simulation results are presented in section 4. The experimental work and results are presented in section 5, followed by the conclusions in section 6.

## 2 Modeling of the Three-Tank System

**Figure 1:**
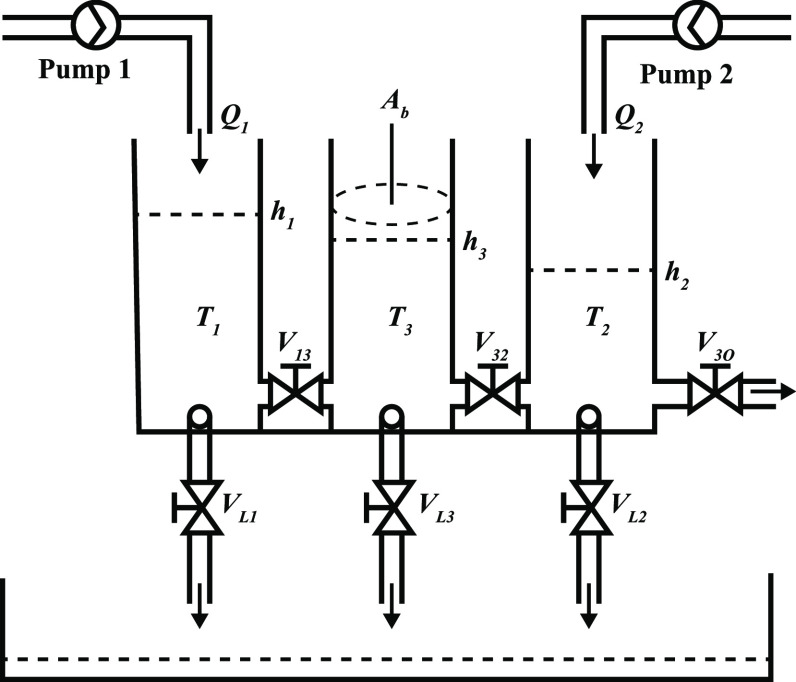
Three-tank system.

The three-tank system consists of tanks named *T*_1_, *T*_3_, and *T*_2_ with the same cross-sectional area *A*_b_, as shown in Figure 1. These cylindrical tanks are connected serially to each other by the cylindrical pipe with cross-sectional area *A*_c_. Liquid is collected in a reservoir and is pumped back into tanks *T*_1_ and *T*_2_ with pumps 1 and 2 to maintain their levels. All of the tanks are equipped with a piezoresistive pressure transducer, which measures the liquid level in the tank.

*Q*_1_ and *Q*_2_ are the flow rates of pumps 1 and 2, respectively. The flow rate provided by the pump is proportional to the DC voltage applied to its motor.

The tanks are equipped with manually adjustable valves and outlets *V*_13_, *V*_32_, *V*_3O_, *V*_L1_, *V*_L3_, and *V*_L2_ for the purpose of simulating clogs as well as leaks. In the tested system, valves *V*_13_, *V*_32_, and *V*_3O_ were open, and the leakage valves *V*_L1_, *V*_L3_, and *V*_L2_ were closed.

The mass balance of the three-tank system is given as follows.^1^
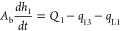
1a
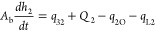
1b
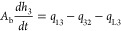
1cwhere *A*_b_ is the cross-sectional area of the tank, *h*_*i*_ (*i* = 1, 2, 3) is the level of the tank *i*, *Q*_*i*_ (*i* = 1, 2) is the flow rate of the pump *i*, *q*_*mn*_ (*m* ≠ *n*) is the flow rate from tank *m* to tank *n*, and *q*_L*i*_ (*i* = 1, 2, 3) is the leakage flow rate of the tank *i*. The flow rates between the tanks and flow rate out from the tank 2 are given by
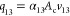
2a
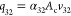
2b
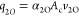
2cwhere α_*ij*_ ∈ [0,1] denotes the outflow coefficient between tank *i*, *j* and out from the tank 2, *A*_c_ denotes the cross-sectional area of the connecting pipe, and *v*_*mn*_ (*m* ≠ *n*) denotes the flow velocity. By assuming that *h*_1_ > *h*_3_ > *h*_2_ and the density of the liquid is constant in the three tanks and using Torricelli’s law based on Bernoulli’s law, the flow velocity between the tanks and out from tank 2 is as follows:
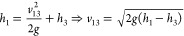
3a
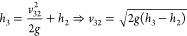
3b
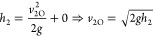
3cInserting eqs 2 and 3 in eq 1, we get the model equations as follows:
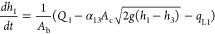
4a
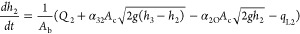
4b
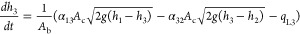
4cwhere *g* is the gravity constant. The linearized state-space model parameters are given by
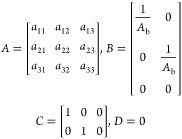
5
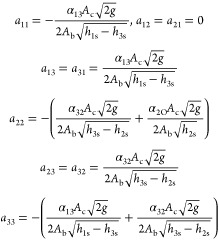
6where *A* is the state matrix, *B* is the input matrix, *C* is the output matrix, *D* is the matrix that describes which inputs affect directly the outputs, and *h*_1s_, *h*_2s_, and *h*_3s_ are the operating points of the three levels, respectively.

## 3 Model Predictive Control for the Three-Tank Pilot System

### 3.1 State-Space Model-Based MPC

As it was possible to develop the detailed physical model of the three-tank system, it was a natural choice to use the linearized version of that model directly with the MPC. The inputs for the MPC are the reference values for the two water levels (*r*) and the measured process outputs for the levels (*y*). The outputs for the MPC are the manipulated variables, two water pump speeds (*u*). The linear state-space system for the MPC is as follows:^12^
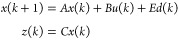
7where *x* are the states, *E* is the disturbance matrix, and *d* are the disturbances.

### 3.2 Regulator

The process is described by the model
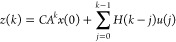
8where *H*(*k* – *j*) are the impulse response coefficients. Using eq 8, the regularized *l*_2_ output tracking problem with the input, the input rate of movement, and the output constraints are formulated as
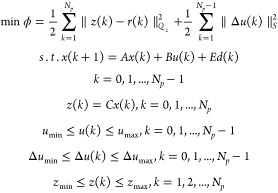
9where Δ*u*(*k*) = *u*(*k*) – *u*(*k* – 1), *N*_p_ is the prediction horizon, *r* is the future target vector, *Q*_*z*_ is the tracking error weight matrix, and *S* is the move suppression factor weight matrix. The vectors *Z*, *R*, *U*, and *D* are defined as
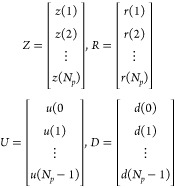
10Then the predictions by the step response model (eq 8) are expressed as
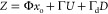
11Φ, Γ, and Γ_d_ are composed as
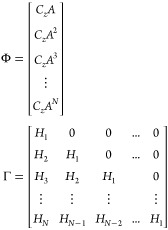
12and
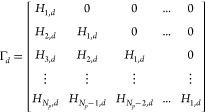
13To clarify, the height of the Λ matrix is one smaller than *N*_p_. Therefore, for the case of *N*_p_ = 6, Λ is composed as
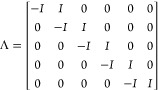
14and
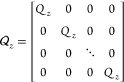
15Compared to the Λ matrix, the height of the matrices *H*_S_ and *M*_*u*_–1__ are same as that of *N*_p_. Therefore, for the case of *N*_p_ = 6, *H*_S_ and *M*_*u*_–1__ are composed as
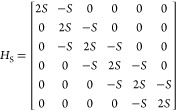
16and
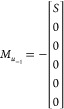
17Then the optimization problem 9 is expressed as
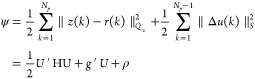
18where
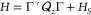
19
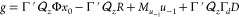
20
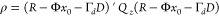
21The state-space-based MPC regulator problem 9 is solved by the solution of the following convex quadratic program
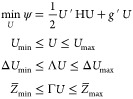
22where
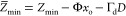
23
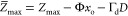
24

In order to remove offset, a control system that can remove asymptotically constant nonzero disturbances is designed. The original system is augmented with a replicate of the constant, nonzero disturbance model.^13^
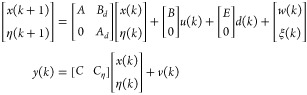
25where η are the integrating disturbance states, and the vectors *w*_k_, ξ(*k*), and *v*_k_ are zero mean white noise disturbances for the states, integrating disturbance states, and the output equation, respectively. In the designed input disturbance model, *B*_d_ = *B*, *A*_d_ is the unit matrix, and *C*_η_ is the zero matrix. For the completeness, the measured disturbances *d* have been included in the augmented model. However, they are 0 in the three tank model. The states and the disturbances are estimated as follows:
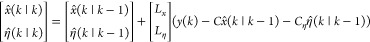
26and the predictions of the future augmented states are obtained by
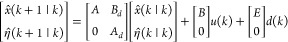
27where *L*_*x*_ and *L*_η_ are the filter gain matrices for the state and the disturbance, respectively. The observability of the augmented system is a necessary and sufficient condition for a stable estimator to exist. If the nonaugmented system (eq 7) is observable and the following condition holds
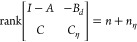
28the augmented system (eq 25) is observable.

If the constraints are not active, the closed loop system is stable and the system model is augmented with a number of integrating disturbances equal to the number of measurements (*n*_η_ = *p*), and there is zero offset in controlled variables.

## 4 Simulation Results

The performance of the developed MPC controller was compared with the PI controller first in the simulation environment. Since the system is relative slow, 1 s was chosen as the sampling time for the simulation. After substituting by the system parameters in Table 1 and discretizing the model, the model is
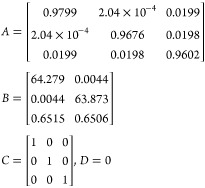
29

**Table 1:** Three-Tank System Parameters

cross-sectional area of the tank (*A*_b_)	0.0154 m^2^
cross-sectional area of the pipes (*A*_c_)	5 × 10^–5^ m^2^
valve opening position (α_*ij*_)	α_*ij*_ = 0.84
maximum flow rate constraint (*Q*_max_)	1.2 × 10^–4^ m^3^/s
maximum level (*h*_max_)	0.63 m
operating point	*Q*_1_ = 5.5 × 10^–5^ m^3^/s
	*Q*_1_ = 3.4 × 10^–5^ m^3^/s
	*h*_1_ = 0.40 m
	*h*_2_ = 0.23 m
	*h*_3_ = 0.31 m

**Figure 2:**
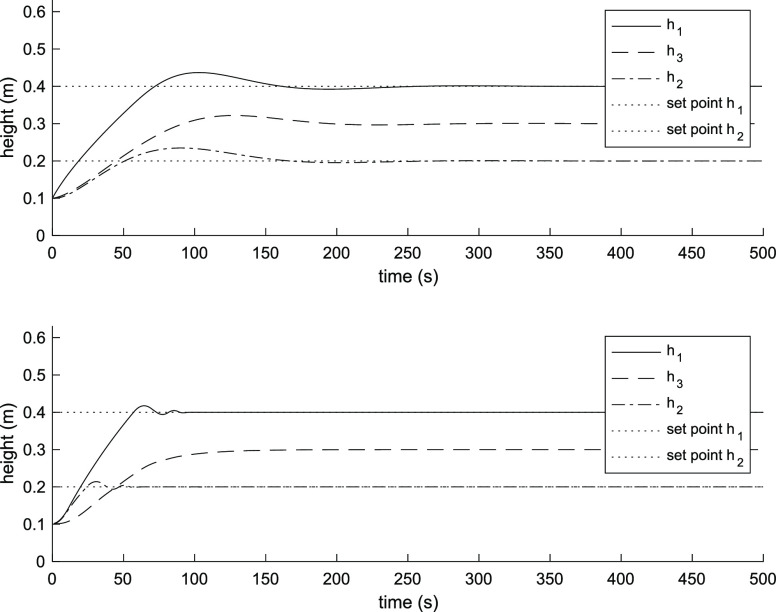
System response using PI controller (above). System response using MPC controller (below).

**Figure 3:**
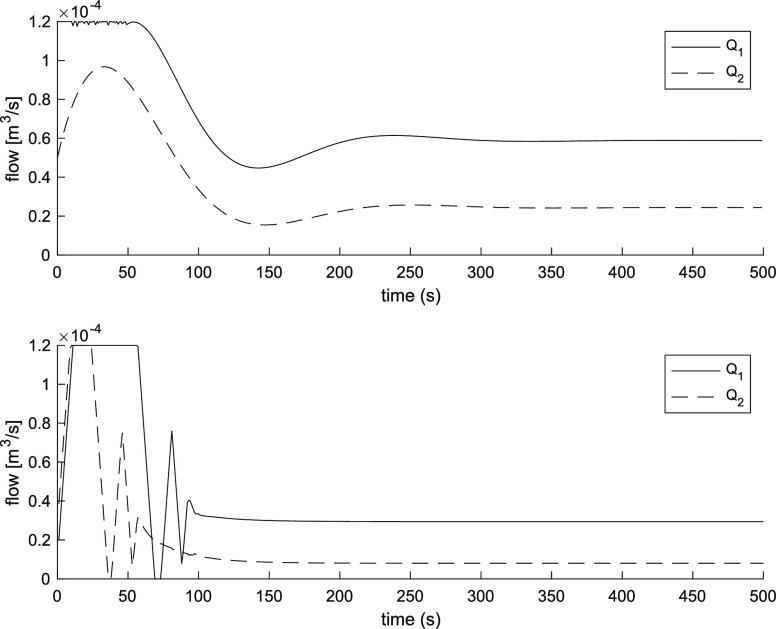
Input flow rates using PI controller (above). Input flow rates using MPC controller (below).

Simulations are carried out for the three-tank system for 500 s. The input limits for the MPC controller were *u*_1,min_ = 0, *u*_1,max_ = 1.2 × 10^–4^, Δ*u*_1,min_ = −1 × 10^–6^, and Δ*u*_1,max_ = 1 × 10^–6^ m^3^/s for the flow rates *Q*_1_ and *Q*_2_. The output limits were *y*_1,min_ = 0 and *y*_1,max_ = 0.63 m for the liquid levels *h*_1_ and *h*_2_. The MPC controller is tuned with
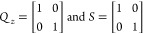
and the prediction horizon *N*_p_ = 40. The parameters of PI controllers were tuned with the Ziegler–Nichols approximate model PID tuning rules^14^ and were detuned with the closed loop method^15^ similar to the one presented by Shamsuzzoha and Skogestad.^16^ Detuning factor used is *F* = 1. The resulting parameters of PI controllers are *K*_p_ = 2.1 × 10^–3^ and *T*_*i*_ = 21 for both liquid levels. In addition, an anti-windup technique was used to eliminate integral term accumulation beyond the saturation limits of the inputs. Figures 2 and 3 show the three-tank level response and input flow rates of the PI controller and the MPC controller. The MPC controller provides a faster response than the PI controller. The first tank level reaches the steady state after 100 s and the second tank after 40 s, taking into consideration the maximum flow rate, while it takes over 175 s for the PI controller for both tanks.

The time-integral performance criteria were used to evaluate the performance of the controllers.^14^ The integral absolute error (IAE) of the MPC controller for the level of tank 1 was 8.76, and for the level of tank 2, it was 1.49. In comparison, the IAE of the PI controller for the level of tank 1 was 12.17, and for the level of tank 2, it was 5.56.

## 5 Experimental Work

### 5.1 System Description

**Figure 4:**
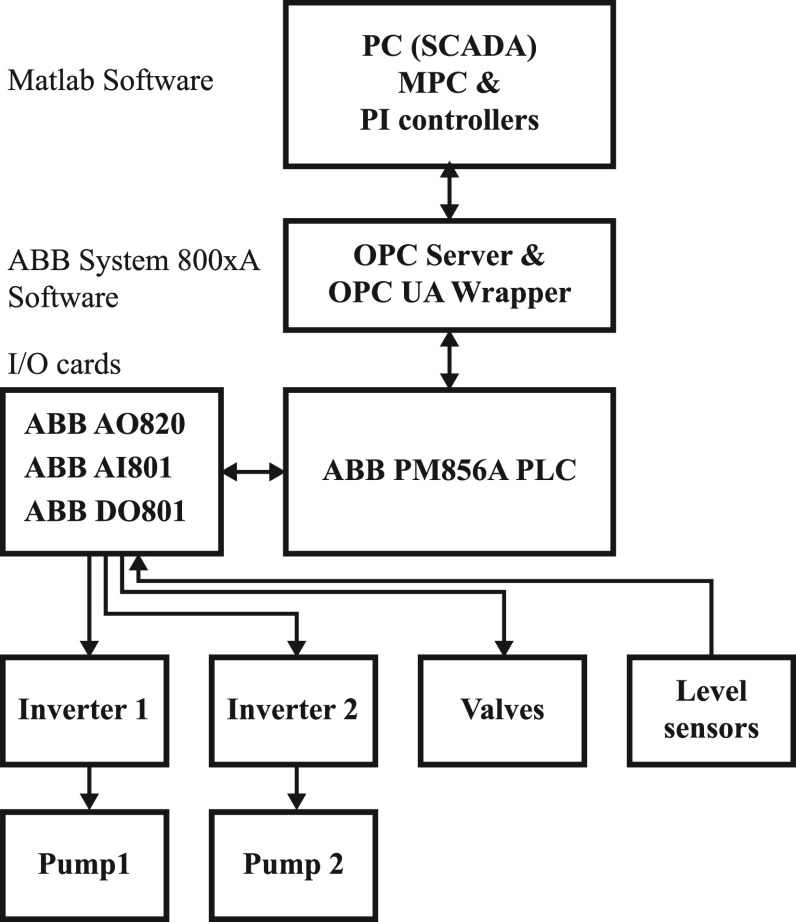
Automation configuration of the three-tank system.

**Figure 5:**
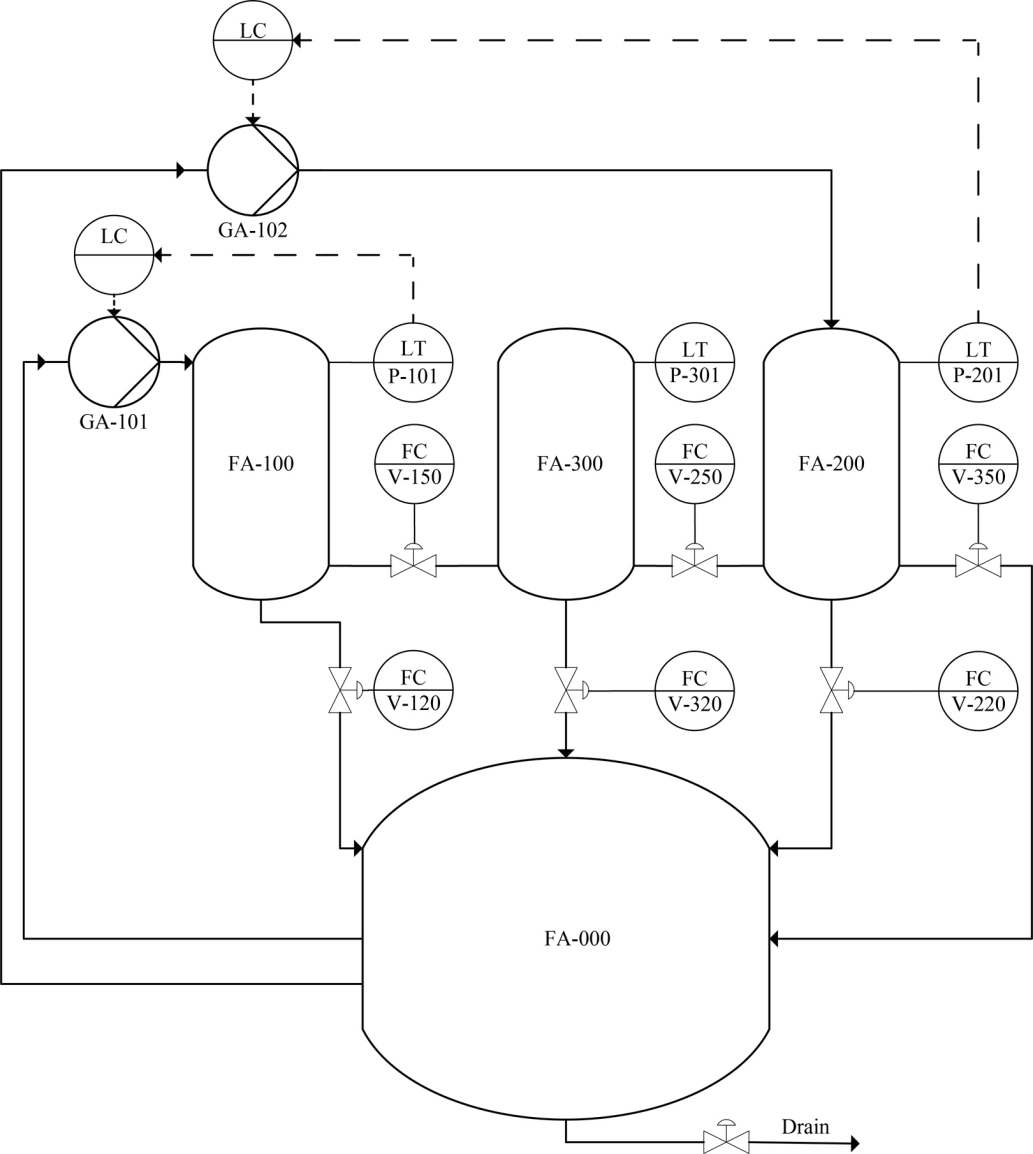
Piping and instrumentation diagram of the three-tank system.

The experimental setup consists of the remote PC running Matlab software, two servers running ABB System 800xA software, the cabin with ABB PM856A PLC and IO cards, and the Amira DTS200 three-tank system represented in Figure 4. Figure 5 shows the piping and instrumentation diagram of the three-tank system. The first two channels of the AO820 card are received for the physical connections of two pumps. Two EPH Electronik inverters (GS24S) have been added between the AO card and the pumps. Table 2 presents the electrical connections of the invertors. Channel 2 receives the voltage from the adapters to provide power for the invertors since they are electrically isolated. Channel 5 sends out the needed signal for the pumps, while channel 8 receives the signal from the analog card (AO820).

**Table 2:** Three-Tank System Parameters

channel	description
1	
2	+ power from 12 V DC adaptor
3	– power from 12 V DC adaptor
4	ground (GND)
5	+ output signal to the pumps
6	– output signal to the pumps
7	+ 10 V bridged to channel 9
8	0–10 V from the AO820 card
9	+ 10 V bridged from channel 9

Level measurements P-101, P-301, and P-201 are wired to AI801 with the RealIO data type. These sensors measure the liquid level and send the 4–24 mA analog signal to AI801 (analog input card).

Six valves in the three-tank system define the input/output arrangements. The valves have electronic actuators. Channels 5–16 of the DO801 card are reserved for six valves: the on and off mode is implemented on each valve. Two SCHRACK relays (RT78725) have been allocated for each valve, one for the on and the other for off mode.

The connections to ABB PM856A PLC and cards are defined in the ABB Control Builder M Professional. Furthermore, the channels of the cards are connected to related variables that are defined in the application. Then the application is downloaded to PLC so that the variables are available on the OPC server. In addition, the Unified Automation UAGateway wrapper/proxy shows OPC servers as folders in its address space.

The MPC controller and PI controllers are implemented on a remote PC through MATLAB software. The communication between PC and PLC relies on the OPC UA wrapper through an Ethernet communication protocol.

### 5.2 Results

Next, the performance of the developed MPC controller was compared with the PI controller in the experimental setup. Due to the OPC UA read and write delays in MATLAB, 2.5 s was chosen as the sampling time for the experimental setup. After the system parameters are substituted in Table 1 and the model discretized, the model is
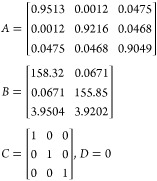
30

**Figure 6:**
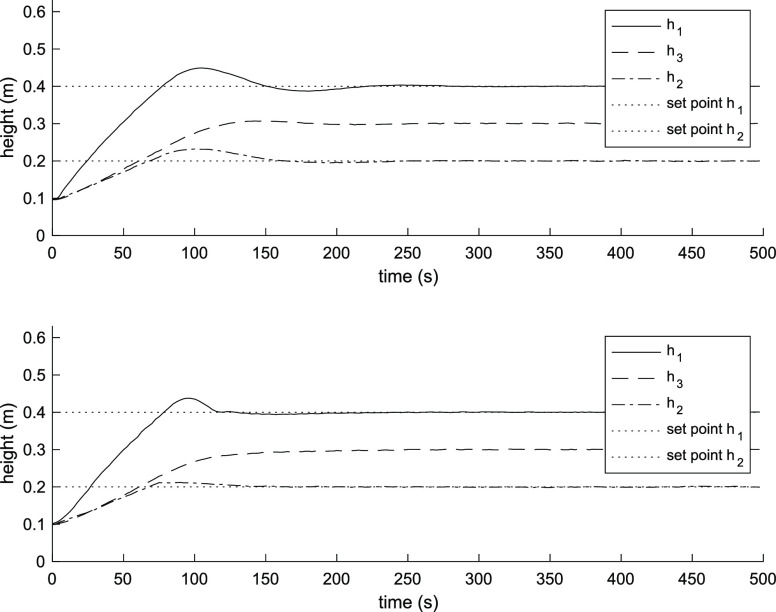
Level responses in experimental setup using the PI controller (top). Level responses in experimental setup using the MPC controller (bottom).

**Figure 7:**
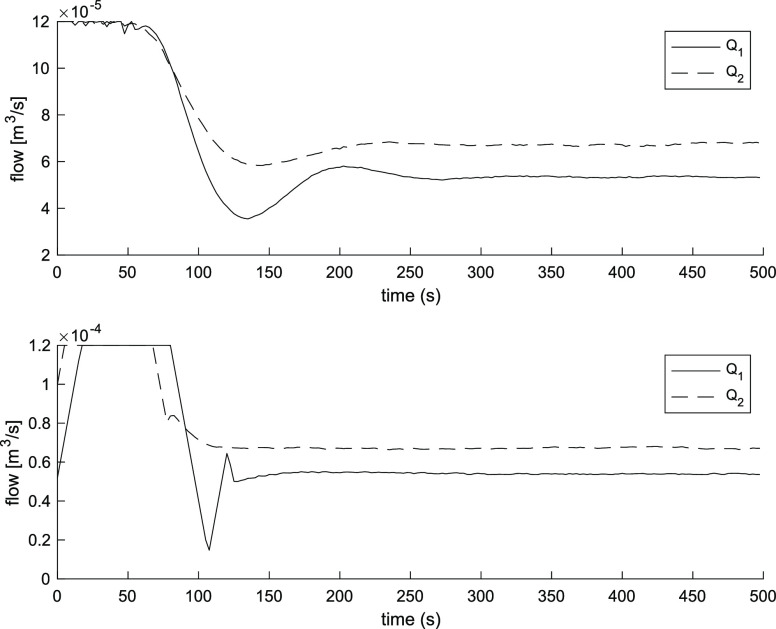
Input flow rates in the experimental setup using the PI controller (top). Input flow rates in the experimental setup using the MPC controller (bottom).

The parameters of the MPC controller and PI controllers are the same as in the simulation. Figures 6 and 7 show the three-tank system level response and input flow rates of the PI controller and the MPC controller. The MPC controller shows a faster response for two tanks, the settling times are about 120 s for both tanks. On the other hand, the settling times for the PI controller are about 200 s for the first tank and 150 s for the second tank. In addition, overshoots for the levels of the tanks using the MPC controller are 9% for the first tank and 6% for the second tank, while for the PI controller, they are 12% for the first tank and 15% for the second tank.

The IAE of the MPC controller for the level of tank 1 was 5.38, and for the level of tank 2, it was 1.79. In comparison, the IAE of the PI controller for the level of tank 1 was 5.54, and for the level of tank 2, it was 2.24.

The MPC and PI controller perform in a similar way when the levels are rising due to the physical limit of 1.2 × 10^–4^ m^3^/s for both flow rates. However, the MPC controller shows the faster settling times for both liquid levels. In addition, the MPC controller can automatically decouple the interactions between the tanks, which results in a lower overshoot in tank 2 in the simulation and in experiment setup. The wilder input variations in the MPC controller are due to the rate of change constraints on the inputs.

## 6 Conclusions

This paper presents the MPC controller for the three-tank system. The simulation results showed the effectiveness of the proposed controller. After that, it has been implemented for the experimental three-tank system setup. The experimental results showed that the settling times are about 120 s for both tanks with the MPC controller, whereas the settling times for the PI controller are about 200 s for the first tank and 150 s for the second tank. In addition, the experimental setup shows how the MPC can be implemented in the remote PC utilizing the new OPC UA communication standard in the industrial automation system.
